# When is an illusion not an illusion? An alternative view of the illusion concept

**DOI:** 10.3389/fnhum.2022.957740

**Published:** 2022-08-31

**Authors:** Brian Rogers

**Affiliations:** Department of Experimental Psychology, University of Oxford, Oxford, United Kingdom

**Keywords:** illusion, perception, seeing, Ames room, perceptual effects, veridical, reality

## Abstract

What is an “illusion”? I would like to argue that (A) there is no coherent and meaningful definition of the word “illusion” and (B) the majority of the things we have previously labelled as “illusions” can be better categorised into three classes of perceptual effects: (i) those that should not be regarded as illusory according to any definition; (ii) those that are simply consequences of “how our perceptual systems work” and (iii) those that are a consequence of using *artificial* or *impoverished* stimulus situations.

## Introduction

What is an “illusion”? It is easy to use the word without thinking whether it has a coherent or meaningful definition. In his last book “Seeing through illusions” (2009), Richard Gregory (2009) defined illusions as *“departures from reality”*—in other words, illusions are situations where what we perceive does not correspond to what is “out there”. The problem with this and similar definitions^1^ is that there are many different things that can be regarded as the “reality” (Rogers, 2017a). For example, the distribution of wavelengths of light reaching our eyes from a particular surface under particular illumination conditions is certainly a “reality” that can be precisely measured with instruments, but no one would regard the fact that the surface colour we perceive does not correspond to that distribution of wavelengths is an illusion. An alternative (and more acceptable) definition of “reality” would be the reflectance characteristic of the surface itself and, once again, those characteristics can be precisely measured with instruments. Under most circumstances, the surface colour we perceive *does* correspond to reflectance characteristics—something that we label as “colour constancy”—and I doubt whether anyone would regard the colour constancy in our perception as an illusion. Does it become an illusion if constancy is not perfect? That sounds arbitrary.

In this article, I would like to argue that: (A) there is no coherent and meaningful definition of the word “illusion” and (B) the majority of the things we have previously labelled as “illusions” can be better categorised into three classes of *perceptual effects*^2^: (i) those that should not be regarded as illusory according to any definition; (ii) those that are simply consequences of *“how our perceptual systems work”* and (iii) those that are a consequence of using *artificial* or *impoverished* stimulus situations.

## The Categorisation of Illusions

**Figure 1:**
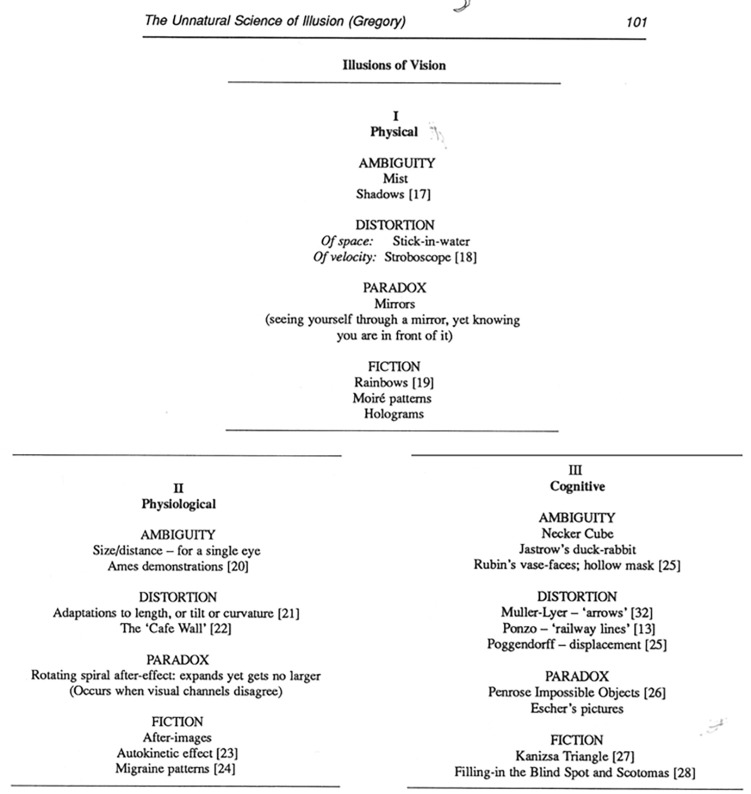
Gregory’s (1966) classification based on the *cause* of the illusion: Physical, Physiological or Cognitive.

Let us start by considering how the things we call illusions have been categorised in the past. In his 1972 book *“The Psychology of Visual Illusion”*, Robinson (1972) categorised illusions according to their perceptual dimension—e.g., size, shape, colour, motion and so on. A similar categorisation was adopted in the chapters of the magnificent *“Compendium of Visual Illusions”* (2017) edited by Shapiro and Todorovic (2017). Although useful, categorisations that are based on perceptual dimensions obscure the fact that there are often similar explanations (in terms of the putative underlying mechanism) across different dimensions e.g., contrast effects, assimilation, normalisation, adaptation and so on. A quite different categorisation was proposed by Richard Gregory (1996) in an article entitled *“The Unnatural Science of Illusions”*. Gregory’s categorisation of illusions is based on putative causes: perceptual effects that have: (i) a “physical” basis; (ii) a “physiological” basis, or (iii) a “cognitive” basis (Figure 1). As someone interested in human (and animal) perception, I do not regard the effects that have a “physical” basis—such as rainbows, moire patterns and bent sticks in the water—as being illusions because they tell us nothing about the workings of our perceptual systems. These effects are a consequence of physics rather than the visual system.

How about the distinction between those perceptual effects that have a “physiological” basis and those that have a “cognitive” basis? I think we can all agree that motion, colour, and size after-effects can be explained in terms of a mechanism such as adaptation. But what about the perceptual effects that have a “cognitive” basis? Gregory argues that the Müller-Lyer and Ponzo illusions are a consequence of applying “size-scaling rules” in the visual system. But are there any “rules” (as opposed to regularities) in the visual system? It could argued that many of the effects described as having a “cognitive basis” are simply those effects that we do not yet understand.

So what is the alternative to the categorisations of illusions based on the perceptual dimension or the underlying cause? I would like to suggest that most, if not all, of the perceptual effects that are typically labelled as illusions can be divided into three classes: (i) those that should not be regarded as illusions according to *any* definition; (ii) those that are consequences of *“just how our perceptual systems work”* and (iii) those that are a product of using *artificial* or *impoverished* stimulus situations.

## Perceptual Effects—An Alternative Perspective

### Situations that should not be regarded as illusory according to any definition

**Figure 2:**
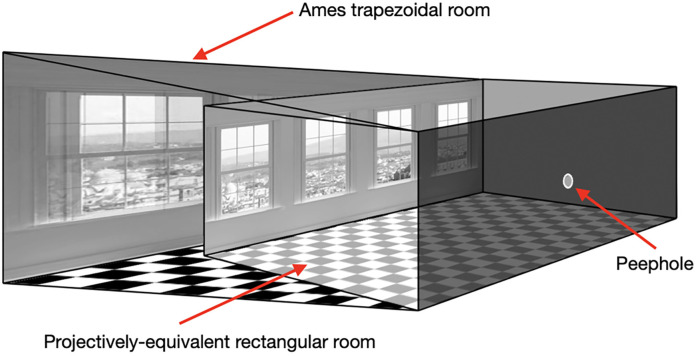
This figure shows the construction of an Ames Room, which is designed so that the pattern of light reaching the “peephole” from the trapezoidal room is the same as that created by a normal rectangular room (from Rogers, 2017b).

My first example of a situation that should not be regarded as illusory according to any definition is the well-known Ames Room. First described by Adelbert Ames in the 1950s, the Ames Room has a trapezoidal shape^3^ with one of the far corners of room being much farther away than the other (Ames, 1955). To construct an Ames Room, the far wall has to be slanting off to the left (in Figure 2) and there also has to be an increase the wall’s height so that the *angular* height of the two corners is the same, when viewed from a particular vantage point (peephole; Figure 2).

The Ames Room is regarded as an illusion because what we see—a normal rectangular room—does not correspond to the “reality”—a trapezoidal shaped room. There is a mismatch between what we perceive and what is “there in the world” and hence, according to the traditional definition, the Ames Room should be categorised as an illusion. But, as Richard Gregory pointed out in 1966 in his book *“Eye and Brain”*:

“*It must look like a normal room if constructed according to strict perspective, and viewed from the* *right position, because the image it creates is the same as for an ordinary room*”.

As a consequence, no seeing machine (biological or man-made) could ever distinguish between the pattern of light created by a properly constructed Ames Room and that created by a normal rectangular room^4^. The correct question to ask is not “why do we see the Ames Room as rectangular” but rather “why do we see *rectangular* rooms as rectangular?” The answer to that question is that there must be perspective information, such as a parallel contours of the floor and ceiling lines, the texture gradients and the shapes and sizes of the back windows, that define the room’s shape. The fact that the room’s “real” shape is trapezoidal is totally irrelevant. You may wish to argue, however, that the Ames Room becomes interesting when there are people standing in the two corners of the room (and I agree). What this shows is that the way we judge size is typically based on relative size so that, in this case, the figure standing in the far corner is seen as smaller, rather than farther away.

**Figure 3:**
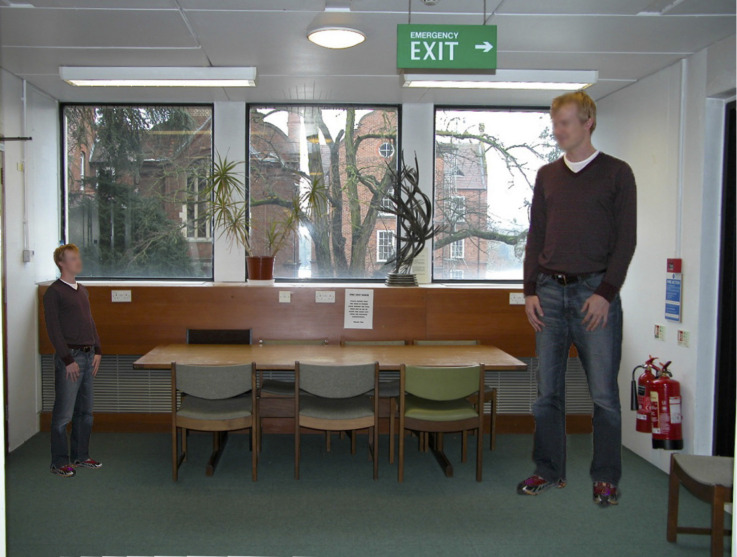
“Ceci n’est pas une Ames room?” (with apologies to Rene Magritte). This is a photograph of a normal *rectangular* room onto which I have superimposed the images of two identical but differently-sized figures (from Rogers, 2017a Figure I10–1).

Another way of making the same point is that we do not need an Ames Room to investigate the relative contributions of perspective and familiar size. Figure 3 shows my own Ames Room with a pair of identical twins standing in the left- and right-hand corners. However, this is not an Ames Room but rather a photo of a normal, rectangular room onto which I have superimposed the images of a pair of identical but different-sized figures into the two corners. This makes the point very clearly that the trapezoidal shape of the original Ames Room is totally *irrelevant—*it could have been circular room or even a flat photograph as long as it creates the same pattern of light at a single viewing position as a rectangular room. What is important is the perspective information the room provides.

It is also important to remember that viewing the Ames Room (or the world) through a peephole is an impoverished perceptual situation. Peephole viewing not only eliminates the binocular information that would normally be available to us but also the motion parallax information that we are able to use when we move around in the world. However, and contrary to my own expectations, our recent experiments have shown that under binocular viewing—and therefore providing binocular disparities to specify the trapezoidal shape of the Ames Room—those disparities are *not* sufficient to override the perspective information specifying the rectangular shape of the room (Rogers, 2021).

As a consequence, I would like to argue that the Ames Room or any other situation that creates the *same* pattern of light at the eye (the optic array) as another real-world scenario: (i) is not an illusion, and (ii) tells us nothing that we did not know or could not find out by looking at the real-world scenario it mimics. I refer to these situations as *facsimiles^5^*.

**Figure 4:**
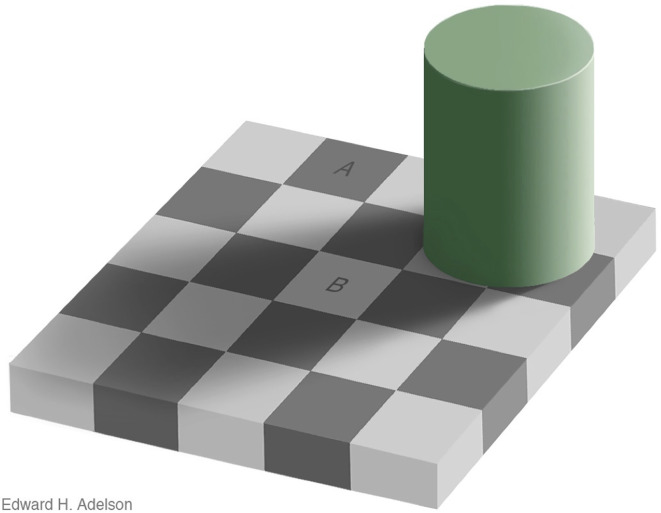
Adelson’s checker shadow illusion.

A second example of a facsimile is Ted Adelson’s checker shadow effect (Figure 4). It has been regarded by many as an illusion because the amount of light coming from one of the dark checks: (A) is the same as that coming from one of the light checks; (B) (this is the “reality”) whereas what we perceive is that: (A) is a dark check; and (B) is a light check. As argued previously, the mistake in categorising this as an illusion arises as a consequence of the particular choice of “reality”. In this case, the reality is assumed to be the amount of light reaching our eyes from a particular surface. But simple physics tells us that the amount of light reaching our eyes from a particular surface tells us *nothing* about the lightness (the reflectance characteristic) of that surface because it is always confounded by the level of illumination. Hence it is an inappropriate basis for determining whether the situation should be categorised as an illusion. Fortunately, but not surprisingly, evolution has provided us with a perceptual system that allows us to correctly judge the lightness of a surface under most conditions of illumination—in other words there is lightness constancy.

A second way to convince yourself that the checker shadow effect is not an illusion is to imagine that you are viewing an actual checkerboard, rather than the photo of the checker board. What are we likely to perceive? Answer: that “A is a dark square and B is a light square” and this perception is fully consistent with the reality of the checkerboard because it is made up of light (high reflectance) and dark (low reflectance) squares. Of course there is conflicting information when the same pattern of light comes from an image on a flat sheet of paper but does that suddenly make our perception “illusory”?

A third example of a facsimile is the viewing of stereo pictures through a stereoscope. Many people, including some of my own students, regard the viewing of pictures through a stereoscope as an illusion because the “reality” is that the two stereo pictures are *flat* but what we perceive is a three-dimensional scene. There is a mismatch between the physical reality and what we perceive. Once again the mistake we make is in choosing one particular definition of reality—the flatness of the individual stereo pictures. The alternative reality is that there are differences between the two stereo pictures—binocular disparities—and evolution has provided us with a perceptual system that allows us to perceive the 3-D structure of world from those disparities. In other words, our perception of a scene in depth is quite consistent with the pattern of disparities between the two images. Our perception is correct (or veridical) rather than illusory. A second way to convince yourself that the viewing of pictures through a stereoscope is not an illusion is to imagine viewing the world through a head-mounted device in which images of the surrounding world are displayed on two small (flat) screens in front of the two eyes, using the signals from two cameras just in front of the head-mounted device. The physical “reality” in this case is that the images displayed on two small screens are flat but we still perceive the depicted scene as three-dimensional. Is this an “illusion”? Would it be an “illusion” if the world were viewed indirectly through a collection of lenses and mirrors? What is important in assessing whether a given perceptual effect is veridical or an illusion is the *availability of information^6^* in the patterns of light reaching our eyes, not the way those patterns of light are created.

There are many more examples of “facsimiles” (or equivalent configurations, Runeson, 1988) that have traditionally been labelled as illusions but, in all cases, I would argue that: (i) they should not be regarded as illusions; and (ii) they tell us nothing that we didn’t know or couldn’t find out by looking at the real-world scenario that the facsimile mimics (Rogers, 2017a). It could be argued that the perceptual effects in the facsimile category are similar to those that are that have a “physical” basis according to Gregory’s classification: i.e., if the pattern of light reaching the eye(s) from a facsimile is the same as that reaching the eye(s) from the scene it mimics, it is a consequence of physics rather than the perceptual system.

### Illusions that represent “just how the system works”

**Figure 5:**
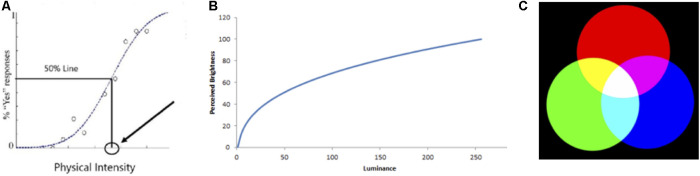
Examples of **(A)** thresholds, **(B)** non-linearities, and **(C)** metamerism.

There is a second group of perceptual effects that can be categorised as *“just how the system works”*. A good example can be seen in a threshold situation (Figure 5A). A very dim spot of light that observers do not report as seeing would not be regarded as an illusion even though there is a mismatch between the reality (a dim spot of light) and what we perceive (nothing). Similarly, we would not regard the non-linearities in the visual system as illusions. We might double the intensity of a super-threshold spot of light (the reality) but what we perceive is only a small increase in the perceived luminance (Figure 5B). There is a mismatch between the reality and what we perceive but we do not regard non-linearities as illusions but rather *“just how the system works”*.

A third example of a situation that we would not regard as an illusion is the mixing of coloured lights—metameric matches. A mixture of long wavelength (red) light and medium wavelength (green) light is perceived as yellow (Figure 5C). We do not regard this as an illusion—a mismatch between the reality (“red” + “green” wavelengths) and what we perceive (“yellow”)—but rather as just how our trichromatic colour system works. Of course, this is obvious to any visual scientist but for anyone who does not know about trichromacy, this demonstration might seem surprising and remarkable. And this gives us a clue about the nature of many of the perceptual effects that others might categorise as illusions: we give the label “illusion” to things that we do not yet understand or cannot explain. Once we have an explanation there is no illusion (at least for vision scientists) —it is “*just how the system works”*. Sadly, we have yet to find a satisfactory explanation of either the Müller-Lyer illusion or the Moon illusion and, as a consequence, we still refer to them and label them as “illusions”.

But what about light and dark adaptation? Is the difficulty in finding a seat in a dark theatre after being outdoors in a bright environment, an illusion? Clearly not. Is the inability to point correctly at a target after wearing prismatic glasses, an illusion? Clearly not. Our perceptual systems have evolved to adjust and compensate for changes in the illumination conditions and unreliability of proprioceptive/motor signals about eye position. But what about the after-effects of prolonged stimulation along a particular sensory dimension such as colour, motion or size? There is certainly a mismatch between the “reality” and what we perceive in each of these situations but for me they are not illusions but rather examples of *“just how the system works”*?

### Artificial, impoverished or ambiguous situations

There is a third class of perceptual effects that, in my view, should not be regarded as illusions: these are situations where there is *inadequate, impoverished* or *ambiguous* information. My argument is that it *has to be true* that if you take away the information that the perceptual system normally uses, our perceptions will not correspond to the reality of the situation. It follows that whatever we discover about the performance of the perceptual system in artificial, impoverished or ambiguous situations may not tell us very much about how we perceive in more normal, real-life situations. For example, are the simple, black-and-white, cartoon-like face drawings that Fantz used in his early experiments the best way to study face perception (Fantz, 1961)? A second example of an impoverished situation comes from the experimental studies that have used short-duration tachistoscopic presentations. The usual justification is that tachistoscopic presentations eliminate the confounding effects of eye movements but they also exclude the additional information that is normally available to us when we view and scan real-world scenes.

**Figure 6:**
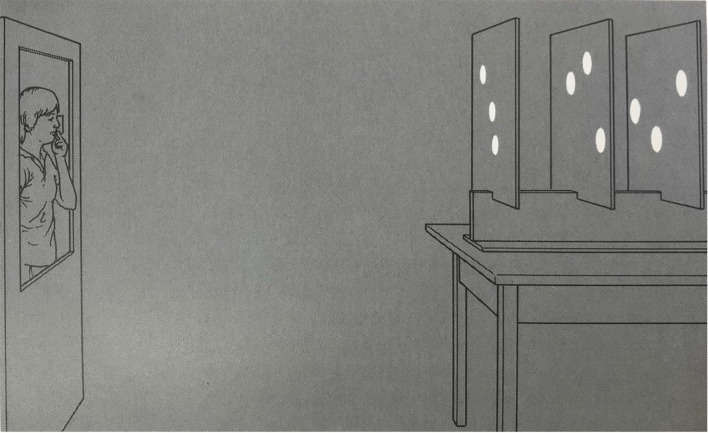
The apparatus used in Rock’s motion parallax experiment in which a monocular observer moves from side-to-side whilst viewing nine luminous discs in an otherwise dark room (from Rock, 1984).

Consider a more complex situation based on my own research on motion parallax as a source of information about the structure of objects and their layout in the surrounding world. A few years after Maureen Graham and I published an article showing that observers can correct perceive the shape of 3-D surfaces when motion parallax is the only source of information (Rogers and Graham, 1979), Irv Rock described an experiment in which he and Deborah Wheeler presented nine luminous discs—three groups of three discs at three different distances from the observer—in an otherwise dark room (Figure 6). Monocular observers were allowed to move their heads from side-to-side through 15 cm and asked whether they saw the three groups of discs at the same or different distances. Their results suggest that many observers did not see the actual 3-D layout of discs.

At first, we were concerned that Rock’s results were contrary to our own findings but it occurred to us that his experimental situation was extremely impoverished—the only visual stimulation was a few isolated flat discs in an otherwise dark room^7^. As a result, it should not surprise us that we fail to perceive the situation correctly given that we have taken away the information normally used by the visual system. We live in a rich world of objects and surfaces, including the ground plane (Rogers, 2020), rather than a world of isolated discs suspended in mid-air, and it is not obvious that we can extrapolate from the latter situation to the former. Hence, the failure to see the true layout of the discs in Rock’s experiment should not be regarded as an illusion but rather as an example of perceptual *failure* due to the use of impoverished stimuli.

**Figure 7:**
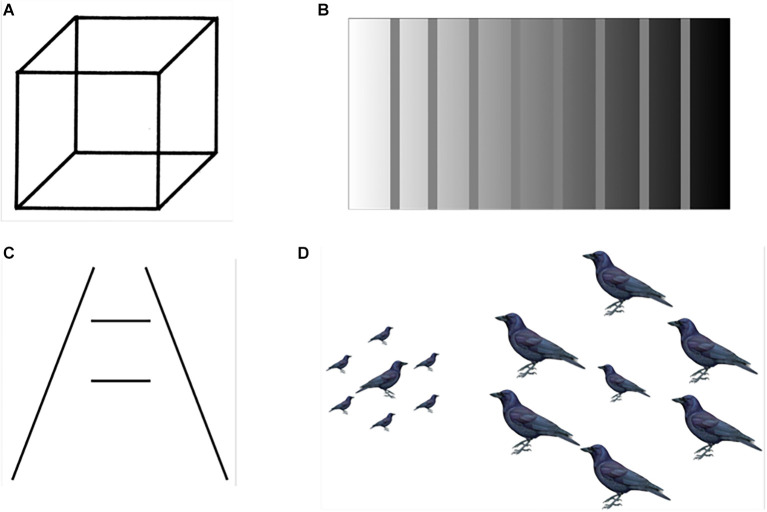
**(A)** Necker cube, **(B)** Simultaneous contrast, **(C)** Ponzo figure, and **(D)** an ecological version of the Ebbinghaus figure.

Many, if not most of the experiments we undertake in the lab use artificial, impoverished or ambiguous stimuli. The justification for using “simple” stimuli such as line drawings, grating patterns and random dot stereograms displayed on computer monitors is that we might be able to generalise from these simple stimuli to more complex real-world situations—a strategy that has been very successful in the natural sciences. But is this the best way of investigating human perception? I would argue that it is not. Let me justify this with a few examples. Take the Necker cube (Figure 7A). Our perception of the wire frame model of a cube is ambiguous—sometimes the cube appears to be facing downwards and to the left but on other occasions it appears to be facing upwards and to the right. Our perception is ambiguous. But why? In 1968 Gibson (1968) wrote: *“The perception is equivocal because what comes to the eye is equivocal”* (p247). But what does this result tell us about the perception of cubes in a real-world environment?

We have evolved and live in a world of surfaces that are typically opaque and because of this there is little or no ambiguity about the 3-D structure of a typical, opaque Necker cube. The outline Necker cube is a fun effect but should not be regarded as an illusion because it is based on an impoverished stimulus in which the information that we typically use is absent. As a consequence, it tells us very little about the normal functioning of our perceptual systems.

A second example of a situation that is often regarded as an illusion is the dramatic contrast effect shown in Figure 7B. Each of the vertical strips reflects the same amount of light to your eye but the left-most strip appears to be a very dark grey and the right-most strip a very light grey. Is this an illusion? The first mistake is to think that the amount of light reflected off a given surface could tell us *anything* about the lightness (reflectance characteristic) of the surface^8^. It cannot because it is always confounded by the intensity of the illumination. Not surprisingly, evolution has provided us with a perceptual system that takes this “into account” (Rock, 1983) and allows us to perceive the lightnesses of surfaces correctly in real-world situations—in other words, there is lightness constancy. Once again, this demonstration tells us very little about our perceptual system because the information that is normally used by the visual system when viewing natural scenes is excluded.

A third example of an effect that has traditionally called an illusion was first demonstrated by Mario Ponzo (1928). The upper of two parallel horizontal lines in Figure 7C (which are of equal length on your screen), appears to be slightly longer than the lower line. The angular (or retinal) size of the lines is the same (if the figure is presented in a frontal plane) but we perceive the two lines to be of different lengths. As a result, there is a mismatch between the reality and what we perceive. But line drawings are necessarily ambiguous because they lack the information about size and distance that is normally present in the world. The converging outer lines of the Ponzo figure could have been created by a pair of truly converging lines located in a frontal plane or a pair of parallel lines that recede into the distance—an ambiguity that is beautifully exploited in Magritte’s painting “The Avenue of Euclid”. In real-world viewing, however, such ambiguities are rare because we live in a world of 3-D structures and surfaces, rather than thin lines.

Once again, the mistake lies in assuming that the angular subtense can tell us something about size in the world. The angular subtense of an object varies as a function of distance and therefore it is an inappropriate way of describing the “reality”. Traditionally, we have assumed that the size of objects in the world has to be calculated by a higher-level process that “takes distance into account” (Rock, 1983). However, the empirical evidence suggests that the perceived size of objects in the real world is more often determined by *relative size* (as can be seen in the Ames Room demonstration—Figure 3), rather than by *“*taking distance into account” or some process of size-scaling. Many, if not all, of the classic geometric “illusions” such as the Müller-Lyer, Ponzo, Hering, Poggendorf and Zöllner effects can be characterized as impoverished stimulus situations and it is worth noting that we do not have a satisfactory explanation for most of these “illusions”.

## Some Thoughts on Todorovic’s (2020) “Augmented Framework for Illusions”

In 2020, Dejan Todorovic’s took on the ambitious task of attempting to answer the question: *“What are Visual Illusions”* (Todorovic, 2020). The central aim of his article was not to provide a satisfactory definition of the illusion concept but rather to put forward *“*An Augmented Framework for Illusions” that could be used to decide whether or not a particular perceptual effect should be given this label. Todorovic’s justification was that:

*“…broad definitions* (of the illusion concept)* are too broad and include phenomena which are* ***not classical illusions****”* (p1132, my emphasis).

Whereas his Augmented Framework was:

*“An attempt to formulate an approach to illusions with a* ***narrower scope***.” (p1132, my emphasis).

To my mind, it is this “narrower scope” that is the weakness of Todorovic’s critique of illusions. He makes the point that in each of the classical, figural illusions—such as the Müller-Lyer, Ponzo, Zöllner, Ebbinghaus, and Ehrenstein-Orbison figures (as well as in simultaneous contrast effects) there is a “*target”* in the figure—for example, the length of the shafts of the Müller-Lyer figure—and a *“context”*—the arrowheads positioned at either end. This distinction between “target” and “context” raises the question of whether the visual system does, or is able to, parse the patterns of light reaching the eye in this way (see Morgan, 1996) but I will put that problem aside for the moment^9^.

According to the augmented framework, a given situation is an “illusion” if a change in the *“context”* affects the perception of an unchanging *“target”*—for example, by changing the arrowheads to orthogonal lines at the end of the Müller-Lyer shafts or by altering the grey shade of the surround in a simultaneous contrast situation. But note that Todorovic’s analysis ignores the fact that the angular size of the shafts in Müller-Lyer figure or the horizontal lines of the Ponzo illusion represent just one particular description of the “reality” of the situation (see the Section “Introduction” above). In the case of the Ponzo illusion, the identical angular substense of the horizontal (“target”) lines might appear to be an appropriate description of the “reality” when the figure is viewed on a flat piece of paper or computer screen (Figure 7C) but not if the the similar lines are part of 3-D scene. In the simultaneous contrast situation, the amount of light reaching the eye from a given surface (“target”) tells us nothing about the lightness of that surface and, once again, it is an inappropriate description of the “reality” of the situation (see Rogers, 2017a). It is also worth pointing out that most of the perceptual effects identified in Todorovic’s restricted framework (e.g., the classical geometric/figural illusions) are effects for which we do not have a satisfactory explanation, as Todorovic himself acknowledges (p1192).

In general, no-one would want to disagree with the general notion that “context” affects our perceptions but this is just a label rather than an explanation of *why* the context might affect our perceptions. When we start to think about the *why* question, it is obvious that the perception of a particular feature—the “target”—cannot be determined by the proximal (retinal) size, shape, luminance, wavelength distributions, disparity, and motion of the “target” alone. In my view, simultaneous contrast effects are better understood as a consequence (an unfortunate by-product?) of the visual processes that have evolved to to provide us with veridical information about the world—e.g., lightness constancy—rather than being labelled as illusions. Once again, this makes the point that there are dangers in using the findings from artificial or impoverished situations as the basis for our understanding of perception.

And the same “why” question applies to other “context” effects. For example, the Ebbinghaus figure (Figure 7D) should be classified as an illusion according to Todorovic’s Framework, because the two central crows (the “targets”) are perceived to have different sizes when one of the crows is surrounded by smaller crows and the other by larger crows (the “contexts”). The same objections apply. First, the angular size of a “target” tells us nothing about its actual size (because angular size varies as a function of distance) and therefore may not be the best way to describe the “reality”. Second, we do not perceive people or objects in a real-world scene to be different in size when they are closer or farther away (despite their different angular sizes) because evolution has provided us with what we refer to as size constancy. In my view, constancies should not be thought of as “add-ons” to correct for the insufficiency of the receptor signals but rather they are the essence of a visual system that has evolved to allow us to (more-or-less) correctly perceive the actual sizes, orientations, motions and the reflectance characteristics of surfaces. Characterising these situations as the “context” that affects the perception of the “target” is misleading.

Todorovic acknowledges that while his augmented framework based on “targets” and “contexts” might be a suitable way to categorise figural illusions and simultaneous contrast effects, it does not cover many other situations—such as metamerism and the Ames Room—that many people regard as illusions. To deal with these additional situations, Todorovic has proposed a set of additional *“criteria”* that have to be fulfilled in order to classify a particular perceptual effect as an “illusion”. These include the *“contextual origin criterion”*, the *“congruency criterion”*, and the *“interaction criteria* (p1157 and p1163).

Take, for example, Todorovic’s treatment of the Adelson’s (1995) Checker Shadow effect (Figure 4). He acknowledges that if we consider a real 3-D scene (of a cylinder standing on a checker board, the situation would not be regarded as illusory—we correctly perceive the reflectance characteristics of the black and white chessboard squares, even in the presence of shadows. However, Todorovic argues that the situation is different when the same pattern of light reaches our eyes from a flat picture. He makes the point that all pictures, paintings and photographs depicted on a flat piece of paper or on a canvas have a dual identity—they could either be a depiction of a real 3-D scene or just a collection of lines and other features on a flat surface (see Gregory, 1966 p169). As a consequence, they provide the observer with *conflicting* information. But note that when the picture or photograph is viewed by a stationary monocular observer and there is no visible texture of the picture’s surface, there is (in the limit) no difference in the pattern of light reaching the eye compared with the same pattern of light from a real 3-D scene. I would argue that because no observer or seeing machine could tell the difference, it does not make sense to categorise our perception as illusory simply because the picture *is* flat. But what happens when there is conflicting information (e.g., viewing a picture with two eyes)? Does our perception suddenly become as an illusion because our perception is inconsistent with *one* particular source of information —the binocular disparities—that indicate that the surface is flat? One could equally argue that our perception of a 3-D scene is *not* illusory because what we see is consistent with the perspective, shading and the other information present in the picture. What is important is the information available in the pattern of light reaching our eyes, not how that pattern of light is created.

For Todorovic, pictures and photographs have two different “*interpretations*”^10^ (p1167) and those pictures and photographs are different from a real scene because they only *“represent”* objects (p1165). Of course, pictures and photographs re-present the same pattern of light to the eye as the real scene but I assume that Todorovic must mean something different by the word “represent”. But what about a display such as a 3-D TV or a head-mounted display? Ideally, such a display presents the observer with exactly the same patterns of light to the two eyes as the real scene. If this is the case, it does not make sense to talk of two alternative “interpretations” because there is no conflicting information. But does that make my perception of the depicted 3-D scene *illusory* because the observer is not perceiving the actual scene?^11^.

Todorovic goes on to make the following very revealing statement:

*“Note also that…classical illusions do not involve seeing 3D* ***where it does not exist****, but rather misperceiving* ***2D features which do exist****, such as size, shape or colour.”* (p1162–3, my emphasis).

According to Todorovic, the use of perspective and shading information in a (flat) trompe-l’oeil work of art is sufficient for it to be regarded as a correct perception (rather than an illusion) but the (limited) availability of perspective information in a classical illusion figure makes it an illusion? Where is the boundary (see Rogers, 2017a; Figure I10–5)?

There are similar problems with Todorovic’s treatment of metamerism. Howard and Rogers (1995, p78) describe metamerism as a consequence of any channelled system which has overlapping sensitivity functions, and this applies to colour vision. Todorovic writes:

*“…unlike classical illusions, metamerism is not based on context effects.”* (p1162).

So that, according to Todorovic’s original (non-augmented) framework, metamerism *should not* be classified as an illusion. However, Todorovic goes on to say:

*Metamerism is another case of a discrepancy between reality and appearance which is not an illusion…”* (p1162) because *“… metamerism fails on both the* ***contextual origin criterion**** and the* ***interaction criteria****.”* (my emphasis, p1162).

Once again, this sounds like a very convoluted way of saying that metamerism is a consequence of the way our channelled, trichromatic system works (Figure 5C). Once we understand the properties of a channelled system, there is nothing illusory about the consequences.

Todorovic’s treatment of the Ames Room is similarly problematic. For example, he writes that:

*“For appropriately positioned observers, both rooms (normal and trapezoidal) will look cuboid but this would be **correct** for the normal room and **wrong** for the Ames Room.”* (p1163, my emphasis).

From this I surmise that that our perception of an Ames Room (without people or other objects in the two corners) should be classified as an illusion according to Todorovic’s framework. If true, this shows the inappropriateness of treating the actual shape of the room as being the appropriate description of the “reality”. A naïve observer might be impressed by the fact that s/he sees an Ames Room as rectangular when its “real” shape is trapezoidal but for a vision scientist there is nothing to tell us that the room is other than rectangular.

However, it turns out that Todorovic is ambivalent about whether the Ames Room should be classified as an illusion. He writes that because *“… this effect does not necessarily fail the interaction criteria”* (p1163), it **can** be classified as an illusion but, on the other hand, because it *“fails the congruency criterion^12^”* (p1163), it **cannot** be classified as an illusion. Where does that leave us? On p1164, Todorovic concludes that *“the Ames Room is not an illusion”*. To me, this sounds like a very convoluted way of answering the question of whether the Ames Room is an illusion. It would be more straightforward to say that if the patterns of light reaching the eye are indistinguishable, it makes no sense to regard one way of creating the pattern of light is an correct perception and another way of creating the same pattern of light is an illusion.

But Todorovic goes on to say that:

*“…because it* (the Ames Room) *fulfils several illusion criteria, it could be regarded as a **near-illusion** on an **illusion in an extended sense.”*** (p1164 my emphasis).

In other words, according to the *augmented framework*, the Ames Room may or may not be an illusion. By invoking these different “criteria” in his augmented framework, Todorovic has effectively restricted the range of things that might be classified as illusions to the classical figural illusions (and simultaneous contrast). But this does not address the questions of (i) *why* we suffer from the effects that *are* categorised as illusions or (ii) *why* other perceptual effects *should not* be categorised as illusions (other than that they fail to satisfy the various “criteria” in the augmented framework).

Overall, I think that Dejan Todorovic has done an excellent job of presenting a vast range of empirical findings, demonstrations, discussion and arguments about the nature of illusions. His article is a real *tour de force*. Moreover, it is impossible to do justice to all the details and subtleties of Todorovic’s article in just a few paragraphs. But having said that, I have very significant reservations about whether his *“Augmented Framework”*, or any other set of criteria, rules or procedures for differentiating between what should be called an illusion and what should not, will help us to answer the question of whether the illusion concept itself is coherent and meaningful.

## Conclusions

What can we conclude? First, I want to argue that there is no satisfactory or meaningful definition of what constitutes an “illusion”. The traditional definition of a *“mismatch between reality and what we perceive”* is just not adequate. Words matter (Rogers, 2022), and we fool ourselves if we think there is a coherent and meaningful way of distinguishing between things we label “illusions” and those we label “veridical”. Second, some of the situations that we label as “illusions” are “facsimiles” —i.e., patterns of light reaching our eyes that are merely copies of some other real-world situation. As a consequence, they cannot tell us anything that we could not learn from investigating the real-world situation the facsimile mimics. Third, the use of inadequate, impoverished or ambiguous stimuli such as lines, dots and grating patterns as well as tachistoscopic presentations, restricted visual fields, flat pictures, isolated depth “cues” and experiments in the dark may tell us about how our perceptual system functions in those restricted environments but maybe very little about perception in the real world. You can call these effects “illusions” if you wish but once we have a satisfactory explanation of a particular aspect of perception (such as the constancies, metameric matches, thresholds, adaptation and non-linearities), we no longer regard those perceptual effects as illusions but rather “just how our perceptual system works”.

As one Reviewer of this article pointed out, the distinction between “impoverished or ambiguous” stimuli and “real-world” scenes represents a continuum rather than an absolute divide. Moreover, perhaps the distinction should be based on the *information* that is used to accomplish a particular task rather than the characteristics of the stimulus. A good example would be Johansson’s point-light demonstrations of biological motion. At a stimulus level, the small group of moving dots might be regarded as a very impoverished input but in terms of information, those patterns of moving dots clearly provide rich information about the particular observer—male or female, old or young—and the nature of their locomotion—walking running, dancing, etc.

My aim in writing this article is not to recommend that we abandon all experiments that use those artificial, inadequate, impoverished or ambiguous stimuli. In some cases, the results can give us insights into how the perceptual system works in more natural environments—e.g., the Ames Room provides further evidence that perspective and relative size matter and the Pulfrich Pendulum effect reveals the role of visual latencies. However, if our only aim is to create situations which merely surprise, amuse or excite us, we are not adding very much to our understanding of perception.

## Author Contributions

The author confirms being the sole contributor of this work and has approved it for publication.

## Funding

This research was conducted with the help of a grant from the Leverhulme Trust (grant/award number: 78176).

## Footnotes

^1^Nick Wade has argued that illusions *“require a yardstick or referen cerelative to which (the illusion) can be assessed”*. For Barbara Gillam, *“the term illusion typically refers to a discrepancy between perceived reality and objective or physical reality”*.

^2^The examples of *perceptual effects* described in this article are all in the visual modality but I think that *perceptual effects* in other modalities such as audition and touch could be similarly differentiated according to this scheme.

^3^Strictly speaking, the room has a hexahedral shape (Day, 1993).

^4^This is not to say that any seeing machine would see the pattern of light as a room (distorted or otherwise) but rather that no seeing machine could ever detect a difference between the patterns of light created by a properly constructed Ames Room and a normal rectangular room.

^5^The dictionary definition of the word “facsimile” is “an exact copy” which I think correctly describes the situations described in this article. I could have used the words “equivalent configuration” but I think this is more restrictive.

^6^I am using the word “information” to refer to what the world offers us and what is available in the pattern of light reaching the eye e.g. the rate of dilation providing information about time-to-contact. But note that once the information used to achieve a particular perceptual task is identified, we are effectively providing a description of the mechanism that is used to extract that information (see Rogers, 2017a). Vision science is all about identifying the relevant information.

^7^Barbara Gillam (personal communication) pointed out that all nine discs subtended the same visual angle which could be regarded as information that all the discs were located at the *same* distance from the observer.

^8^I have discussed the problems of identifying the “reality” with which our “perception” might be compared in Chapter 10 of Shapiro and Todorovic’s *Oxford Compendium of Visual Illusions* (Rogers, 2017a).

^9^Todorovic acknowledges this when he writes: *“However, although subjects should easily identify the targets as such, it turns out that they are not able to process some the features independently from some types of contexts.”* (p1165).

^10^But who or what is doing the “interpreting”?

^11^i.e., in other words, the 3-D TV or head-mounted display creates a facsimile for the observer.

^12^With respect to the congruency origin, *“the physical attributes of the to-be-compared pairs of target objects are congruent, that is, they are either both distally and proximally equal.”* (p1157).
